# Supplementary material for: HIVprotI: an integrated web based platform for prediction and design of HIV proteins inhibitors
Source: J Cheminform. 2018 Mar 9;10:12. doi: 10.1186/s13321-018-0266-y (PMC5845081; doi:10.1186/s13321-018-0266-y)
Supplement: Supplementary file 2 — Additional file 2. Source code of HIVProtI web server. [file 13321_2018_266_MOESM2_ESM.zip › HIVprotI_Source-code/hivprotI/jsmol/jmol-flot.htm]

|  |  |
| --- | --- |
|  |  |

Plot 1:
none
temperature
straightness(p)
straightness(c)
structure
  
Plot 2:
none
straightness(p)
straightness(c)
straightness(r)
structure
  
Ramachandran Plot
Plot 1 vs 2
DSSP
print
  
  
  
show:
data(JSON)
data(TAB)
axes
options

This page and jmol-flot-energy.htm illustrate how the Flot graphing package can be used with Jmol to depict molecular data graphically. The Flot package is quite flexible and very easy to work with. For these pages I've adapted it a bit -- the modified flot JavaScript file is in the flot subdirectory. Mostly I added a hover delay option and the option to hover over a vertical or horizontal slice rather than exactly on a point. For the Ramachandran Plot, I added the option to have major tick marks drawn in a darker color.
